# 3D coral-like nitrogen-sulfur co-doped carbon-sulfur composite for high performance lithium-sulfur batteries

**DOI:** 10.1038/srep13340

**Published:** 2015-08-20

**Authors:** Feng Wu, Jian Li, Yafen Tian, Yuefeng Su, Jing Wang, Wen Yang, Ning Li, Shi Chen, Liying Bao

**Affiliations:** 1 School of Material Science and Engineering, Beijing Institute of Technology, Beijing, 100081, China; 2 School of Chemistry, Beijing Institute of Technology, Key Laboratary of Cluster Science, Ministry of Education, Beijing Key Laboratary of Photoelectronic/Electrophotonic Conversion Materials, Beijing, 100081, China; 3 Collaborative Innovation Center of Electric Vehicles in Beijing, Beijing, 100081, China

## Abstract

3D coral-like, nitrogen and sulfur co-doped mesoporous carbon has been synthesized by a facile hydrothermal-nanocasting method to house sulfur for Li–S batteries. The primary doped species (pyridinic-N, pyrrolic-N, thiophenic-S and sulfonic-S) enable this carbon matrix to suppress the diffusion of polysulfides, while the interconnected mesoporous carbon network is favourable for rapid transport of both electrons and lithium ions. Based on the synergistic effect of N, S co-doping and the mesoporous conductive pathway, the as-fabricated C/S cathodes yield excellent cycling stability at a current rate of 4 C (1 C = 1675 mA g^−1^) with only 0.085% capacity decay per cycle for over 250 cycles and ultra-high rate capability (693 mAh g^−1^ at 10 C rate). These capabilities have rarely been reported before for Li-S batteries.

Li**-**S batteries with the advantages of significantly high theoretical energy densities (2600 Wh kg^−1^), natural abundance and environmental tolerance, show great potential to satisfy the high-energy demands of electric vehicles (EVs) and sustainable energy**-**storage systems^1^,^2^,^3^,^4^. Despite these advantages, several inherent problems still have plagued the practical application of Li**-**S batteries, such as low sulfur utilization, poor cycle life and limited rate performance. These deficiencies are attributed to the poor ionic and electronic conductivities of sulfur and its various discharge products (Li_2_S_*x*_, *x *= 1–8), the dissolution of the intermediate polysulfides, and the slow reaction kinetics of the sulfur reduction^5^,^6^,^7^,^8^. Thus, it is of great signifacance to develop an appropriate method to fabricate high performance Li-S cathode materials with enhanced rate ability and prolonged cycle life.

A variety of strategies have been explored to address the challenges mentioned above. For example, sulfur is combined with highly electronic conductive substrates (micro/mesoporous carbon^9^,^10^, carbon nanofibers^11^,^12^, polymer material^13^,^14^, graphene^15^,^16^,^17^, etc.) to improve the electrical contact. Among these substrates, mesoporous carbons have been proved to be especially promising^10^,^18^,^19^,^20^,^21^, since the sulfur can be uniformly encapsulated inside the porous structures, which favours the capture of the intermediate polysulfides and rapid transport of Li^+^ in the electrode, thereby suppressing the shuttle phenomenon. However, issues still remain in rate capability and cycling stability of these composites, and high reversible capacity has not been successfully achieved during high rate cycling.

Recently, Wang *et al.*^22^,^23^ reported that nitrogen doping in mesoporous carbon can effectively promote the chemical adsorption of sulfur atoms on oxygen-containing functional groups. Other researchers have fabricated nitrogen-doped carbon/sulfur composites^24^,^25^,^26^ showing rapid charge-transfer ability, as surface modification with nitrogen atoms greatly enhances the electrochemical reactivity and electronic conductivity of the carbon matrices. In addition, sulfur-doped porous carbon is also receiving increasing attention for energy conversion and storage. Since sulfur-doping can change the charge state of the neighbouring carbon atoms, the as-doped carbon materials exhibit enhanced adsorption ability^27^,^28^ and catalytic activity^29^,^30^. Moreover, when sulfur and nitrogen are simultaneously incorporated into the carbon matrix, synergistic effects can occur due to the highly active multiple doping elements. The most studied property of the sulfur and nitrogen dual doped carbon materials is their enhanced electrocatalyst ability^29^,^31^,^32^,^33^. The dual activation of carbon atoms can produce more active sites and increase their activity, therefore, exhibit superior oxygen reduction reaction (ORR) activity than the mono-doped carbons. Furthermore, the dual doped carbon materials have also been widely applied in the fluorescent^34^,^35^, electrical^36^ and energy-storage^37^ areas. As so far few reports of dual doped carbons exist in the field of Li-S batteries, this inspired us to exploit the beneficial influences of the doped heteroatoms in the Li-S systems.

Herein, in order to take advantage of the synergistic effect of heteroatoms co-doping and a 3D porous structure, we apply a coral-like, nitrogen and sulfur co-doped mesoporous carbon (labelled as CNSMC) with convenient conductive framework as a promising sulfur container for high-performance Li-S batteries. By the facile synthesis method, a three-dimensionally continuous and spatially interconnected carbon branch network was formed in our CNSMC, and ordered mesopores were homogenously embedded with a nitrogen content of 5.3 wt% and a sulfur content of 4.1 wt%. Benefitting from the strong chemical adsorption ability, convenient transport pathway for ions and electrons, and high electronic conductivity of the carbon framework, the as-fabricated CNSMC/S composites have exhibited excellent rate performance with superior cycling stability.

## Results

### Synthesis and characterizations of the CNSMC

**Figure 1:**
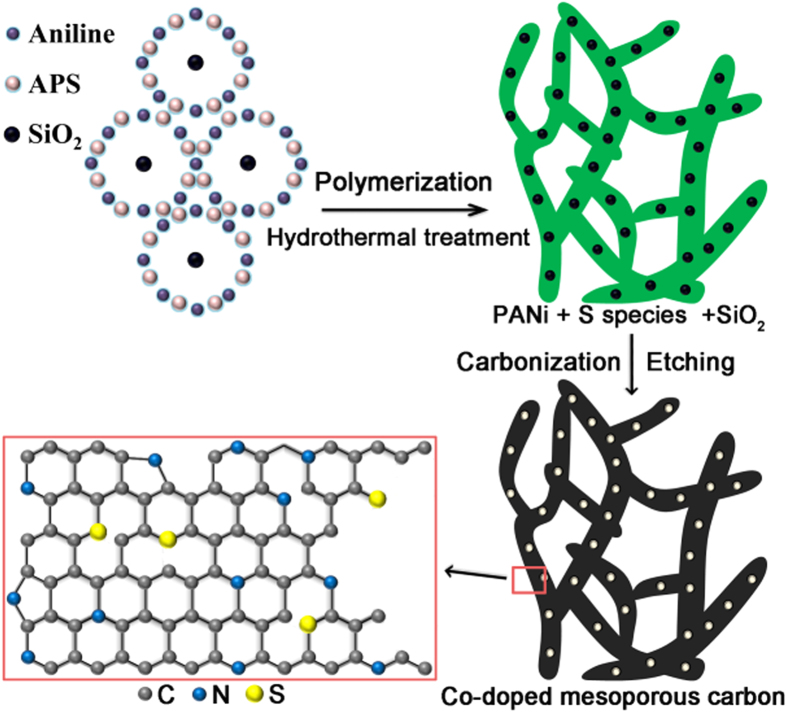
Synthesis of the CNSMC. Preparation of the coral-like, N and S co-doped mesoporous carbon.

**Figure 2:**
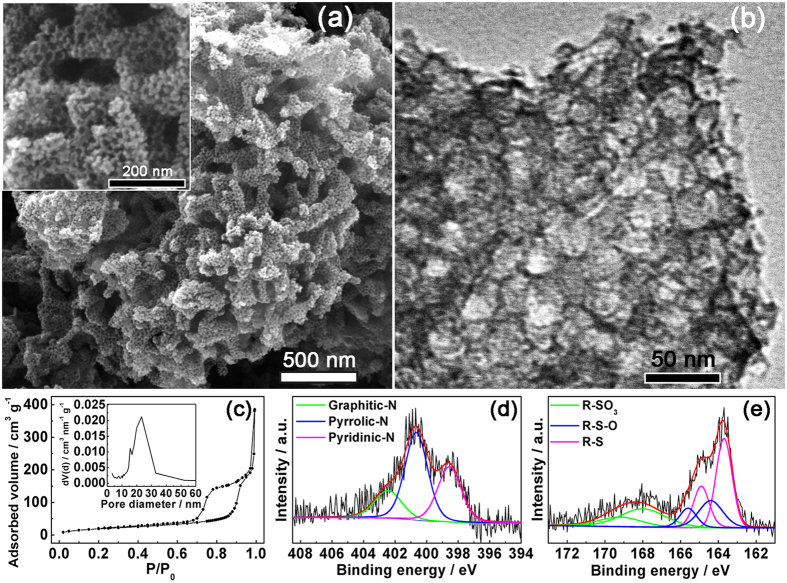
Characterizations of the CNSMC. (**a**) SEM and (**b**) TEM images, (**c**) Nitrogen sorption isotherms (Inset : BJH pore size distribution), High resolution N1s (**d**) and S2p (**e**) XPS spectra of CNSMS.

A hydrothermal-nanocasting method was adopted to grow the 3D self-supported, coral-like, nitrogen and sulfur co-doped mesoporous carbon material. Polyaniline nanofibers with a high N content (15.2 wt%) and excess ammonium peroxydisulfate (APS) oxidazing agent were selected as precursors, and SiO_2_ nanoparticles served as hard templates to form porous structure^38^,^39^, as illustrated in Fig. 1. The scanning electron microscopy (SEM) image in Fig. 2a shows that CNSMC has a hierarchical mesoporosity composed of 3D cross-linked carbonaceous branches. The large numbers of mesopores within the CNSMC can effectively encapsulate sulfur and therefore make it have an intimate contact with the highly conductive carbon matrix. Furthermore, the void space in this carbon framework can offer a large electrode/electrolyte interface and greatly facilitate the Li^+^ diffusion and migration in the material, which is very important for rapid charging/discharging of the electrode. The transmission electron microscopy (TEM) image in Fig. 2b reveals that large numbers of ordered mesopores, which originate from the SiO_2_ templates, are homogeneously distributed within CNSMC. To further examine the porous structure of CNSMC, the nitrogen sorption isotherms were measured (Fig. 2c). The CNSMC displays a pore volume of 0.591 m^3^ g^−1^ (able to hold up to 55 wt% sulfur) and possesses mesopores peaked at about 23 nm. This result agrees well with the observations from the TEM image.

Elemental analysis (EA, Supplementary Table 1) of the CNSMC shows a substantial presence of both the nitrogen and sulfur content, which is 5.0 wt% and 4.6 wt%, respectively. The nature of nitrogen and sulfur species at the surface of CNSMC was investigated by X-ray photoelectron spectroscopy (XPS). The XPS survey spectrum of the CNSMC (Supplementary Fig. 1) shows peaks for C1s (284.6 eV), N1s (400.5 eV), O1s (531.5 eV) and S2p (164.5 eV), confirming the existence of elements detected by EA. The nitrogen concentration on the surface of the CNSMC is determined to be 5.3 wt% by XPS (Supplementary Table 1), which is quite close to the EA result. The N1s peak of CNSMC can be resolved into three components centered at 398.6, 400.7 and 402.4 eV (Fig. 2d), corresponding to pyridinic-N, pyrrolic-N, and graphitic-N doped in the carbon matrix^40^. More than 81.3 at.% of N species in CNSMC are planar, including 48.9 at.% pyrrolic-N and 32.4 at.% pyridinic-N, which are believed to promote the chemical adsorption of sulfur atoms to oxygen functional groups^22^,^23^. As for sulfur, its surface concentration obtained from XPS is 4.1 wt%, which is also close to the value obtained by EA. Analysis of the surface chemistry of CNSMC shows that sulfur is mainly in thiophenic (R–S, 163.6 eV, 47.1 at.%) and sulfonic acid (R-SO_3_, 167.8 eV, 29.4 at.%) configuration (Fig. 2e)^27^,^28^,^30^. The thiophenic-S (sulfur atom bonded with carbon directly by a C–S bond) is believed to make the carbon matrix positively charged, thereby increasing its affinity to adsorb polysulfides^27^,^28^. Above all, the XPS results confirm that we have successfully produced N, S co-doped carbon material by the simple *in-situ* synthesis method.

The CNSMC was also analysed by powder X-ray diffraction in the wide-angle region (Supplementary Fig. 2). From the most relevant characteristic peaks at 2θ = 23 and 43°, it is clear that the CNSMC consists of graphitized carbon, which can greatly affect its electronic conductivity^41^. The Raman spectrum of the CNSMC (Supplementary Fig. 3) shows D- and G-Raman scattering peaks at 1330 and 1592 cm^−1^, respectively^42^. The calculated peak intensity ratio (I_D_/I_G_) is 0.977, also indicates that there is considerable graphitized carbon in the CNSMC^43^.

### Morphology and conductive structure of the CNSMC/S composite

**Figure 3:**
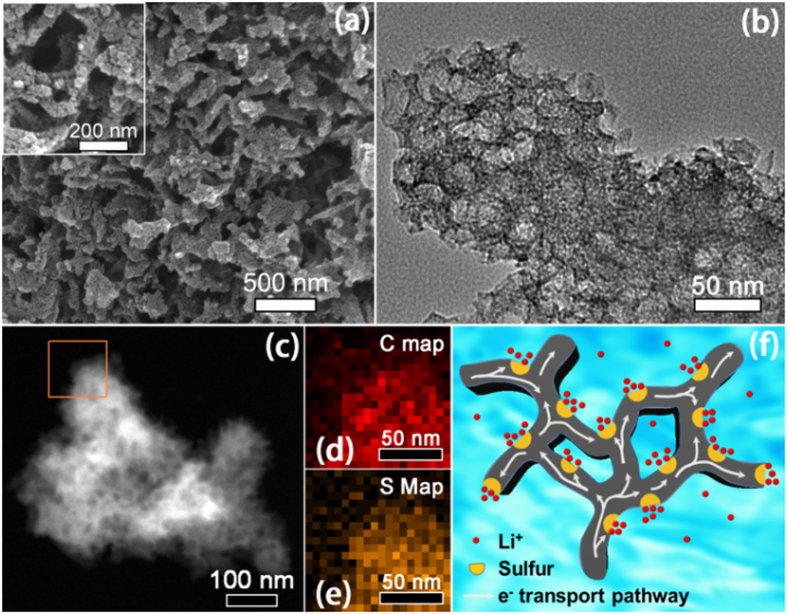
Morphology and conductive structure of the CNSMC/S composite. (**a**) SEM, (**b**) TEM and (**c**) Dark-field TEM images of the CNSMC/S composite. Elemental mapping images of carbon (**d**) and sulfur (**e**) corresponding to the area outlined by the orange square in (**c**). (**f**) Schematic diagram of the CNSMC/S composite with effective conductive pathway.

The CNSMC was further loaded with sulfur via a typical melt-diffusion strategy to form C/S composite^5^. The sulfur content of this composite is about 54 wt%, as determined by thermogravimetric analysis (TGA, Supplementary Fig. 4), and this is close to the EA result (Supplementary Table 1). The homogeneous dispersion of sulfur in the CNSMC matrix is confirmed by SEM, HRTEM, dark-field TEM and the corresponding elemental mapping images (Fig. 3a-e). This composite shows no obvious structural change compared with the pristine CNSMC and most of sulfur is well dispersed in the ordered mesopores without bulk S particles observed. This is also consistent with the XRD pattern, which shows that the diffraction peaks for sulfur become much weaker in the composite (Supplementary Fig. 2). The weak sulfur peaks in the XRD pattern can be attributed to the small content of the crystalline state sulfur. As the sulfur within CNSMC is close to the theoretical maximum adsorption content of its pores, a small number of sulfur may be modified on the outer space of the mesopores when it undergoes co-heat treatment with CNSMC. This external sulfur may leave the pores and re-crystallize into a nano-crystalline state at the conclusion of the heat treatment. Overall, the highly cross-linked C/S network favors intimate contact among the absorbed sulfur, the carbon matrix and the electrolyte, therefore greatly facilitates the high conductivity of Li^+^ and electrons in the electrode (see schematic diagram in Fig. 3f).

### XPS analysis of the chemical adsorption ability of the CNSMC

**Figure 4:**
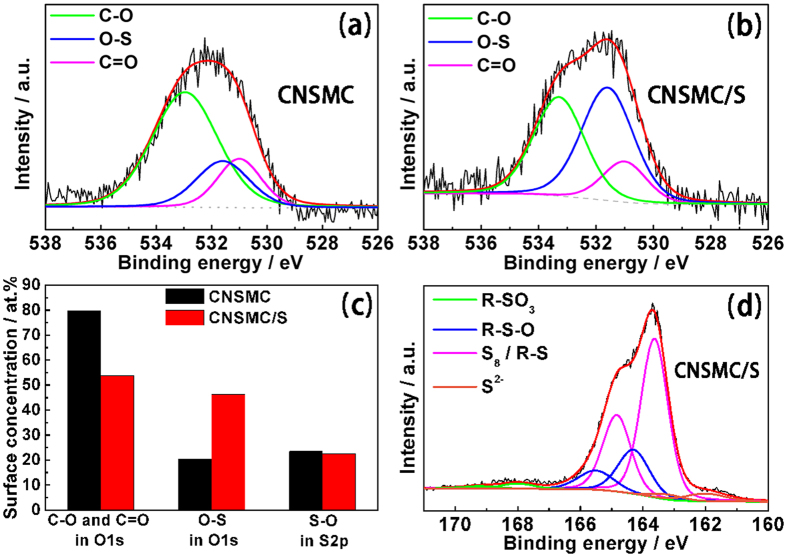
XPS analysis of the chemical adsorption ability of the CNSMC. High resolution C1s XPS spectra of **(a)** CNSMC and **(b)** CNSMC/S composite. **(c)** Changing of the surface concentration of carbon-oxygen species and sulfur-oxygen species in CNSMC and CNSMC/S composite. **(d)** High resolution S2p XPS spectrum of CNSMC/S composite.

As revealed by XPS, the CNSMC with high surface oxygen concentration (9.3 wt%, Supplementary Table 1) is rich in oxygen functional groups, which is common for hydrothermal carbonaceous materials^44^. It is known that the carbon atoms become more positive as the neighboring doped nitrogen and sulfur atoms are more electronegative^27^,^29^. This change of the charge state of the carbon atoms causes the nearby oxygen functional groups to be polarized and activated to anchor sulfur by covalent bonding^22^,^23^,^45^. To investigate the surface chemistry after sulfur incorporation and further elucidate the chemical adsorption ability of the co-doped carbon matrix, a detailed XPS analysis of the O and S species is presented for both the CNSMC and CNSMC/S composite. As shown in Fig. 4a,b, the O1s spectra of both CNSMC and CNSMC/S composite consist of three peaks at 531, 531.6, and 533 eV, corresponding to species of C = O, O–S and C–O (C–O-C, C-OH), respectively^45^,^46^. However, compared with CNSMC, the intensities of all peaks for carbon-oxygen species in CNSMC/S are lower, while the peak corresponding to O–S bond is much higher. The calculated atomic ratio of O–S in CNSMC increases from 20.3 to 46.3 at.% in CNSMC/S (Fig. 4c and Supplementary Table 2). This great change indicates that many chemical bonds between the activated oxygen groups and sulfur were formed after heat treatment of CNSMC with sulfur. Figure 4d displays the S2p spectrum of the CNSMC/S composite. The atomic ratio of S-O species (164.4 eV)^45^,^46^ in CNSMC/S is slightly decreased from 23.5 to 22.5 at.% (Supplementary Table 2), as compared with the CNSMC (Fig. 2e). However, considering that the sulfur content in CNSMC/S (54 wt%) is much larger than that in CNSMC (4.1 wt%), it can also be concluded that a considerable fraction of the sulfur is effectively combined with oxygen functional groups on the carbon surface. The above results show that oxygen functional groups in CNSMC are highly activated in interacting with sulfur, as the electronic structure is changed by the nearby doped N and S atoms. Therefore, besides the shortened and effective conductive pathway, the strong chemical adsorption ability of the CNSMC matrix to sulfur is also expected to contribute to the improved electrochemical performance of the CNSMC/S composite.

### Electrochemical performance

**Figure 5:**
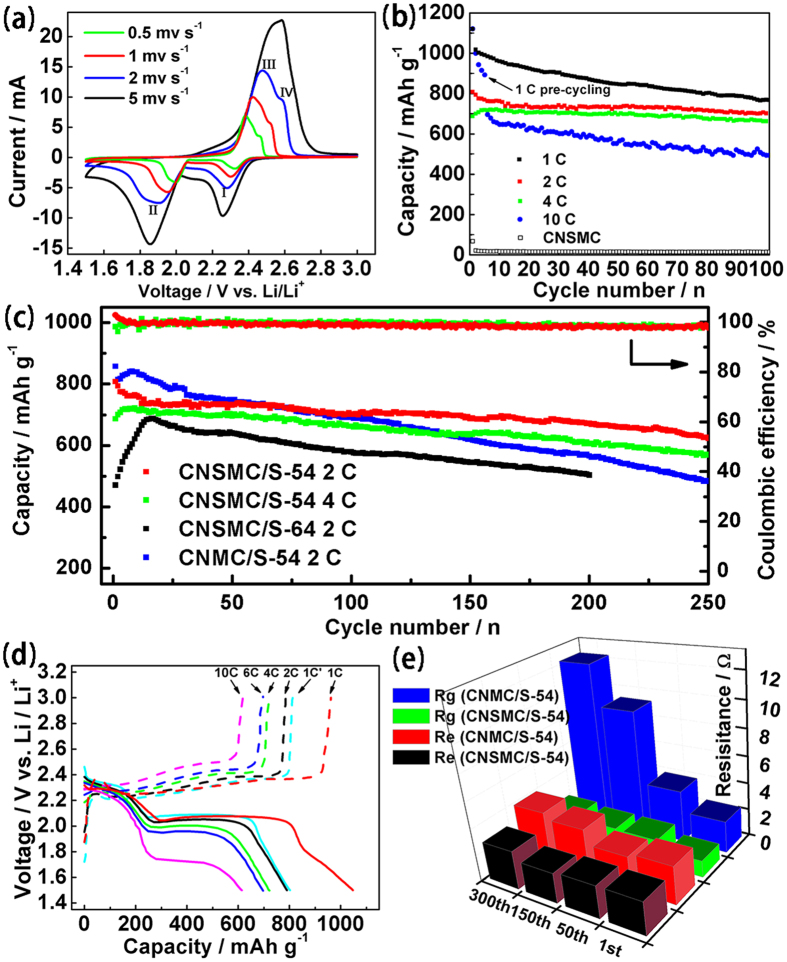
Electrochemical performance. (**a**) Cyclic voltammograms curves with increasing scan rates of the CNSMC/S-54 composite. (**b**) Cycle performance at different current rates of the CNSMC/S-54 composite. (**c**) Prolonged cycle performance and coulombic efficiency of the CNSMC/S and CNMC/S composites (**d**) Galvanostatic charge/discharge profiles at different current rates of the CNSMC/S-54 composite. (**e**) EIS parameters of the CNSMC/S-54 and CNMC/S-54 composites during different cycles.

To explore the electrochemical advantages of this CNSMC/S composite (denoted as CNSMC/S-54), half-cells with metallic lithium anodes were assembled and evaluated. Supplementary Fig. 5 presents the low scan rate CV curves of the composite electrode for the first, second, fourth and eighth cycle. Two pairs of sharp redox peaks are observed due to the typical multiple reactions of sulfur with lithium. None of the peaks show obvious change during cycling, demonstrating the cycling stability of the electrode. Figure 5a also shows the CV curves with increasing scan rates. The peak current and potential each exhibit polarization behaviour with the increasing of scan rate. However, except for peak IV (corresponding to the conversion of Li_2_S_4_ to Li_2_S_8_/S)^1^,^3^, which overlaps with peak III at very high scan rate, complete and well separated peak shapes can still be observed even when the scan rate is as high as 5 mv s^−1^, demonstrating the superior rate ability and excellent reversibility of this C/S composite.

The electrochemical performance of the CNSMC/S-54 composite under different current densities is illustrated in Fig. 5b. The electrochemical capacity is calculated on the mass of sulfur, and it can be seen that the doped S in CNSMC contributes negligible capacity in the tested voltage range. At current rates of 1, 2 and 4 C (1 C = 1675 mA g^−1^), initial reversible capacities of 1120, 805 and 684 mAh g^−1^ are obtained. Furthermore, when the composite is fully activated by pre-cycling at 1 C for 5 cycles, a remarkable initial capacity of up to 693 mAh g^−1^ can be achieved even at the ultra-high rate of 10 C (16.75 A g^−1^). After 100 cycles, the composite shows a capacity retention of 91.7% at 4 C and a high reversible capacity of 478 mAh g^−1^ at a rate of 10 C, demonstrating its outstanding rate performance.

It is known that the cycling stability of the Li-S batteries is seriously restricted by the highly solubility of the lithium polysulfides. Moreover, when tested at high current rates, this problem can be more challenging due to the insulating nature of elemental sulfur and its discharge products. However, based on the strong adsorption ability and effective conductive pathway of the CNSMC matrix, stable cycle performance and high coulombic efficiency of the CNSMC/S composite are successfully achieved, as illustrated in Fig. 5c. After 250 cycles, discharge capacities of 621 and 567 mAh g^−1^ at 2 and 4 C are still maintained of the CNSMC/S-54 composite, with average coulombic efficiencies of 99.2 and 98.7%, respectively. In comparison with the capacity retention of 77.1% at 2 C, this composite shows an even higher capacity retention of 78.9% at 4 C, corresponding to a very small capacity decay of only 0.085% per cycle. Moreover, the stable Q_low_/Q_up_ ratio^47^,^48^ (ratio of the capacity of the lower plateau to the upper plateau, Supplementary Fig. 6M) and well preserved conductive structure of the cycled material (Supplementary Fig. 7) give further evidences of the enhanced adsorption capability, rapid conduction ability and good structural integrity of the CNSMC supporting matrix. Remarkably, Due to the advantages discussed above, the C/S composite with higher sulfur content (64 wt%, denoted as CNSMC/S-64, confirmed by TGA in Supplementary Fig. 4) also shows considerable cycling stability at high current rate of 2 C (Fig. 5c). When calculated based on the total mass of the composite, the reversible capacity of 322 mAh g^−1^ after 200 cycles is still very impressive compared with other C/S composites reported in the literatures (Supplementary Table 3).

The CNSMC/S-54 composite was also subject to cycling at increasing C-rates to evaluate its robustness (Fig. 5d and Supplementary Fig. 8). After an initial discharge capacity of 1043 mAh g^−1^ at 1 C, stable reversible capacities of 787, 719, 695 and 612 mAh g^−1^ are observed when the C-rate is switched to 2, 4, 6 and 10 C, respectively. The stability of the electrode is also evidenced by the recovery of most of the original capacity (799 mAh g^−1^) when the C-rate is reduced back to 1 C, and the voltage hysteresis almost fully disappears (curve 1 C’ in Fig. 5d).

In order to determine the influence of the doped S species, coral-like mesoporous carbon doped only with nitrogen (labelled as CNMC) was also fabricated by a similar procedure as CNSMC. Compared with CNSMC, CNMC has a similar nitrogen doping level (6.1 wt%), however, the sulfur content within CNMC is negligible (0.5 wt%, as shown in Supplementary Table 1). For comparison purposes, the CNMC/S composite with 54 wt% sulfur loading was also fabricated and its cycle performance is shown in Fig. 5c. The CNMC/S-54 composite can deliver more original capacity than CNSMC/S-54 composite for the first 60 cycles when tested at 2 C. The higher initial capacity of the CNMC/S-54 composite is perhaps due to the slightly higher conductivity of the CNMC to its counterpart^49^. However, CNMC/S-54 shows severe capacity decay in subsequent cycles. The reversible capacity of the CNMC/S-54 composite drops to only 484 mAh g^−1^ after 250 cycles, corresponding to a capacity retention of only 56.5%, which is much lower than that of the co-doped C/S composite (77.1%). Moreover, the average coulombic efficiency of CNMC/S composite is also decreased to 98.5% (Supplementary Fig. 9), implying that without the aid of sulfur functional groups, the adsorption ability of the carbon matrix to polysulfides is decreased, resulting in a more serious overcharge phenomenon.

In order to gain insight into the roles of the doped-S species in suppressing the diffusion of polysulfides, electrochemical impedance spectroscopy (EIS, Supplementary Fig. 10) was performed on both the CNSMC/S-54 and CNMC/S-54 composites cycled at 2 C. Figure 5e displays the R_e_ and R_g_ values of the two composites during different cycles derived from the equivalent circuit as shown in Supplementary Fig. 10. R_e_ (associated with the intercept of the semicircles and the real axis of the Nyquist plot) represents the electrolyte resistance, which will gradually increase alone cycling, as the continuous dissolution of the polysulfides increases the viscosity of the electrolyte and then suppress the transport of the lithium-ions^50^. R_g_ (corresponds to the semicircle in the middle frequency range of the Nyquist plot) represents the resistance caused by the continuous deposition of Li_2_S/Li_2_S_2_ film on the electrode^51^,^52^. The R_e_ and R_g_ values of the CNSMC/S-54 composite remain constant (the R_e_ value is even a little lower after 150th cycles) during the long-term cycle process. This is strong evidence that the CNSMC matrix is very helpful in inhibiting the dissolution of the polysulfides, thereby reducing their irregular deposition on the electrode. In contrast, the R_e_ and R_g_ values of the CNMC/S-54 composite keep growing during the cycle process, especially the R_g_ value, which increases from 2.33 Ω in the first cycle to as high as 12.4 Ω in the 300th cycle. This phenomenon indicates that without the aid of the S species, the CNMC matrix shows weaker ability in trapping the polysulfides within its mesoporous structure. Thus, the Li_2_S/Li_2_S_2_ film gradually and irreversibly deposits on the electrode, causing the suppression of the lithium-ions transportation^53^.

## Discussion

The electrochemical results indicate that the co-doped N and S species play an important role in increasing the adsorption ability of the carbon matrix. Their proposed functions are as follows: the doped N and S atoms (in the forms of pyridinic-N, pyrrolic-N and thiophenic-S species) act as electron-withdrawing atoms due to their higher electronegativity than C atoms, causing the nearby C atoms and C–O functional groups (carbonyl, epoxide and hydroxyl) to be polarized and more active in anchoring sulfur and polysulfides. This enhanced chemical adsorption ability of the carbon matrix can facilitate tight adhesion of sulfur during sulfur loading and uniform re-deposition of sulfur during the charging process, thus increasing the electrical contact of the composite and restricting the loss of electro-active materials from the electrode. Moreover, as the carbon-oxygen groups are highly polarized and active due to N and S doping, the sulfur bonded to these groups may also trap the partly-delithiated polysulfides (LiS_x_^−^) through S-S single bonds^22^, thereby suppressing the diffusion of polysulfides that formed in cycle process.

The doped S in sulfonic group (R-SO_3_) may function in anchoring polysulfides by a different way. The negatively charged oxygen ions can be effective in anchoring Li^+^ ions by electrostatic interaction, and these positively charged Li^+^ ions can further trap the negatively charged S_*x*_^2−^. Therefore, the sulfur atom in sulfonic group, which is fixed with three oxygen ions, acts as a strong adsorption site for the polysulfides^23^. Above all, the binding affinity of the carbon matrix is greatly enhanced by the doped N, S species, thereby improving the cycling stability of the CNSMC/S composite.

In conclusion, a novel nitrogen and sulfur co-doped and 3D structured mesoporous carbon material has been synthesized through an *in-situ* constructing strategy for sulfur encapsulation. The as-fabricated composite electrodes exhibit excellent cycle stability at very high current rate. The excellent electrochemical performance can be attributed to the following: (1) the main doped species (pyridinic-N, pyrrolic-N, thiophenic-S and sulfonic-S) increase the affinity of carbon surface to polysulfides and therefore suppress their diffusion; (2) the coral-like framework of CNSMC provides a convenient conductive pathway to ensure the effective and rapid utilization of sulfur; (3) the CNSMC with structural stability acts as a robust supporting matrix to ensure the integrality of the electrode configuration during high C-rate cycling. Further experiments are on-going to understand the overall mechanisms of the co-doped system and to increase the loading and adsorption abilities by tailing the pore size distribution and element doping content of the carbon matrix. The promising electrochemical performance we have observed so far provides clear evidence of the success of our strategy to optimize the mesoporous conductive framework and enhance the surface chemistry by heteroatoms co-doping. Considering the outstanding features discussed above, we believe that our synthetic strategy can be applied not only in advanced Li-S batteries, but also for other promising energy-storage devices.

## Methods

### Synthesis

CNSMC was synthesized as follows: typically, aniline was first dispersed in an aqueous dispersion of 24 nm SiO_2_ nanoparticles (Ludox TM40) in 2 M HCl solution. The suspension was kept at room temperature while the oxidant (ammonium peroxydisulfate, APS), Fe(NO_3_)_3_⋅9H_2_O, and Co(NO_3_)_2_⋅6H_2_O were added with vigorous stirring. The mass ratio of aniline: SiO_2_: APS: Fe(NO_3_)_3_⋅9H_2_O: Co(NO_3_)_2_⋅6H_2_O = 1: 1: 2.28: 0.01: 0.0025. The mixture was stirred for 24 hours to allow the polymerized polyaniline to uniformly mix and cover the SiO_2_ particles. The suspension of SiO_2_, nitrogen-containing polymer, sulfur species (reaction products of APS) and transition metals was transferred to a Teflon autoclave, sealed and treated at 180 °C for 12 h. The resulting pre-heated product was dried at 80 °C, followed by heat treatment to 900 °C and cooling down to room temperature under a nitrogen atmosphere without duration time. The resulting dark solid was treated with 4 M ammonium hydrogen fluoride solution and a 1 M HCl to remove the SiO_2_ template and the metal catalyst, and was then thoroughly washed in deionized water.

The sample doped with nitrogen only (CNMC) was fabricated by a similar procedure as CNSMC, except that the polymerized product (SiO_2_, nitrogen-containing polymer, sulfur species and transition metals) was repeatedly washed before hydrothermal treatment to remove the sulfur-containing species.

The CNSMC/S and CNMC/S composites with 54 wt% sulfur content were prepared following a melt-diffusion strategy. The prepared CNSMC or CNMC and sublimed sulfur (Alfa Aesar) (45:55 w/w) were sealed in a polytetrafluoroethylene container filled with argon gas. The mixture was then heated at 155 °C for 10 h. The CNSMC/S-54 composite was obtained after the mixture cooled down to room temperature. CNSMC/S-64 composite was prepared by a similar procedure using a different C/S ratio (30:70 w/w).

The tap densities of the prepared CNSMC, CNSMC/S-54 and CNSMC/S-64 are 0.15, 0.27 and 0.36 g cm^−3^, respectively.

### Characterization

The surface morphology of the as-prepared samples was obtained by scanning electron microscopy (SEM, FEI Quanta 250) and transmission electron microscopy (TEM, FEI Tecnai F30). Nitrogen adsorption-desorption isotherms were measured at 77 K with an Autosorb-iQ_2_ (Quantachrome) analyzer. The pore size distribution was calculated by the Barrett–Joyner–Halenda (BJH) method, using nitrogen adsorption data. Elemental analysis was carried out using an Elementar Vario EL cube. X-Ray photoelectron spectroscopy (XPS) data were obtained with a PHI Quantera electron spectrometer using Al_Kα_ radiation. X-ray diffraction (XRD) measurements were performed using a diffractometer (Rigaku) with a Cu_Kα_ radiation. Raman spectrum was measured on a spectrometer (Renishaw-2000) with an excitation laser beam wavelength of 532 nm. The thermal gravimetric analysis (TGA) was performed using a thermal analyzer (6200 EXSTAR) at a heating rate of 10 °C/min under nitrogen flow.

### Electrochemistry

The C/S composite cathode slurry was produced by mixing 70 wt% C/S composite, 20 wt% acetylene black and 10 wt% PVDF binder in N-methyl-2-pyrrolidinone. After stirring, the uniform slurry was cast onto aluminum foils and then dried at 50 °C for 24 h. The electrodes were cut into disks with a diameter of 11 mm. The sulfur loading densities on the electrode were about 0.8 and 1.2 mg cm^−2^ for the CNSMC/S-54 and CNSMC/S-64 composites, respectively. Coin-type (CR2025) cells with Li foil as the counter electrode were assembled in an argon-filled glovebox. The electrolyte used was 1,3-dioxolane (DOL) and 1,2-dimethoxyethane (DME; 1:1 v/v) with 1.0 M bis-(trifluoromethane)sulfonimide lithium (LiTFSI) and 0.2 M LiNO_3_. Coin cells were tested at various currents within a voltage range of 1.5–3.0 V vs. Li/Li^+^ using LAND electrochemical station (Wuhan). Cyclic voltammograms were recorded on a CHI 650D electrochemical workstation (Shanghai Chenhua) between 1.5–3.0 V vs. Li/Li^+^. AC impedance was also measured using the CHI 650D electrochemical workstation. The AC amplitude was ±5 mV, the frequency range was applied from 100 kHz to 0.01 Hz. The measurement of the cycled cells was conducted at full charged state.

## Additional Information

**How to cite this article**: Wu, F. *et al.* 3D coral-like nitrogen-sulfur co-doped carbon-sulfur composite for high performance lithium-sulfur batteries. *Sci. Rep.* **5**, 13340; doi: 10.1038/srep13340 (2015).

## Supplementary Material

Supporting information
