# Supplementary material for: 3D coral-like nitrogen-sulfur co-doped carbon-sulfur composite for high performance lithium-sulfur batteries
Source: Sci Rep. 2015 Aug 20;5:13340. doi: 10.1038/srep13340 (PMC4542155; doi:10.1038/srep13340)
Supplement: Supporting information [file srep13340-s1.doc]

Supplementary Information

**3D coral-like nitrogen-sulfur co-doped carbon-sulfur composite for high performance lithium-sulfur batteries**

Feng Wu^1,3^, Jian Li^1^, Yafen Tian^2^, Yuefeng Su^1,3,*^, Jing Wang^1,3^, Wen Yang^2,*^, Ning Li^1^, Shi Chen^1,3^ & Liying Bao^1,3^

^1^ School of Material Science and Engineering, Beijing Institute of Technology, Beijing, 100081, China.

^2^ School of Chemistry, Beijing Institute of Technology, Key Laboratary of Cluster Science, Ministry of Education, Beijing Key Laboratary of Photoelectronic/Electrophotonic Conversion Materials, Beijing, 100081, China.

^3^ Collaborative Innovation Center of Electric Vehicles in Beijing, Beijing, 100081, China.

* E-mail: [suyuefeng@bit.edu.cn](mailto:suyuefeng@bit.edu.cn); wenyang@bit.edu.cn

1. Supplementary Figures.


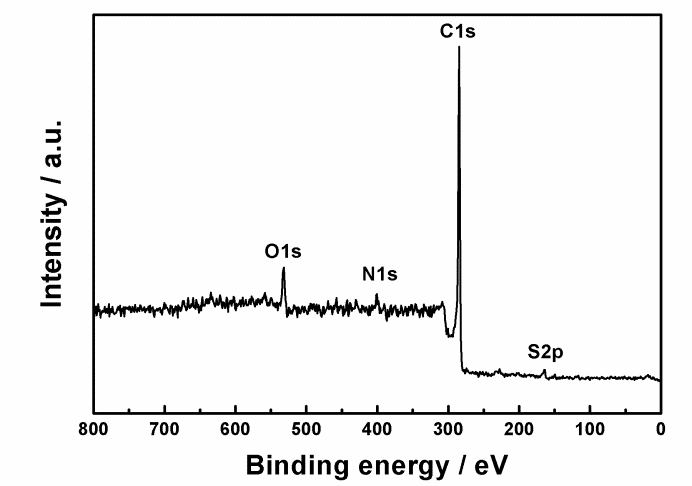


Supplementary Figure 1 **|** XPS spectrum shows the C1s (284.6 eV), N1s (400.5 eV), O1s (531.5 eV) and S2p (164.5 eV) peaks of the CNSMC.


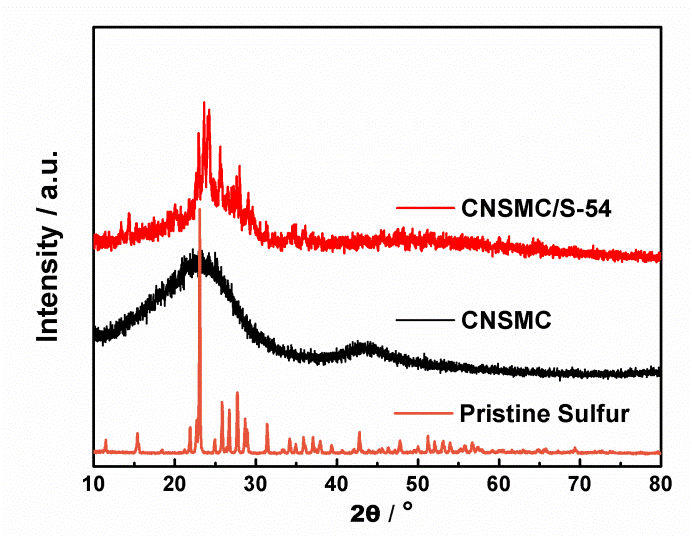


Supplementary Figure 2 | XRD patterns of pristine sulfur, CNSMC and CNSMC/S-54 composite.


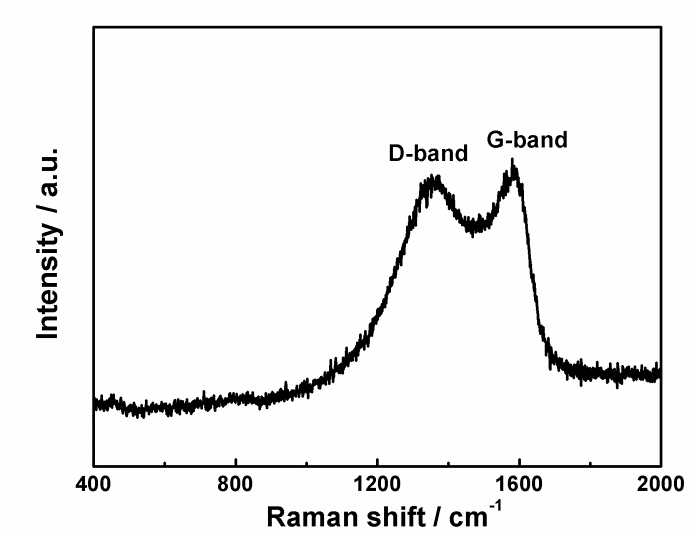


Supplementary Figure 3 | Raman spectrum of the CNSMC.


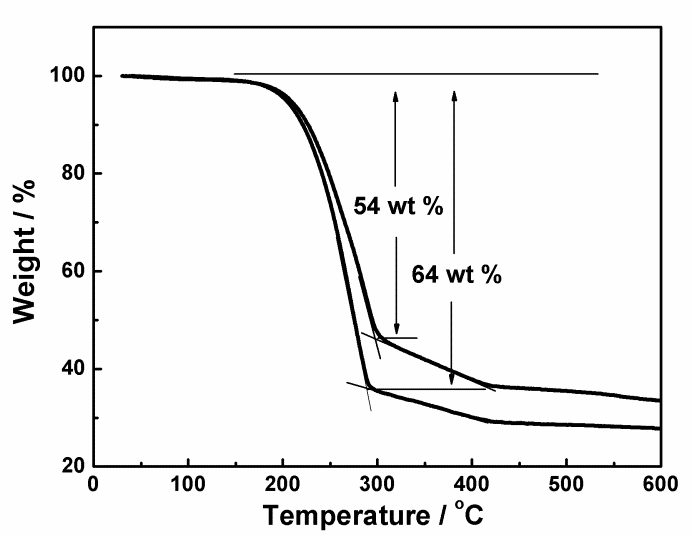


Supplementary Figure 4 | TGA results of the CNSMC/S composites. These composites show a weight loss of 54 and 64 wt% between 170 and 300 °C, which is attributed to the release of sulfur confined within the carbon matrix. A second weight loss process between 300 and 410 °C can also be observed. This phenomenon is commonly seen in the polymer/sulfur composites, and is probably due to the decomposition of large numbers of functional groups in the CNSMC.


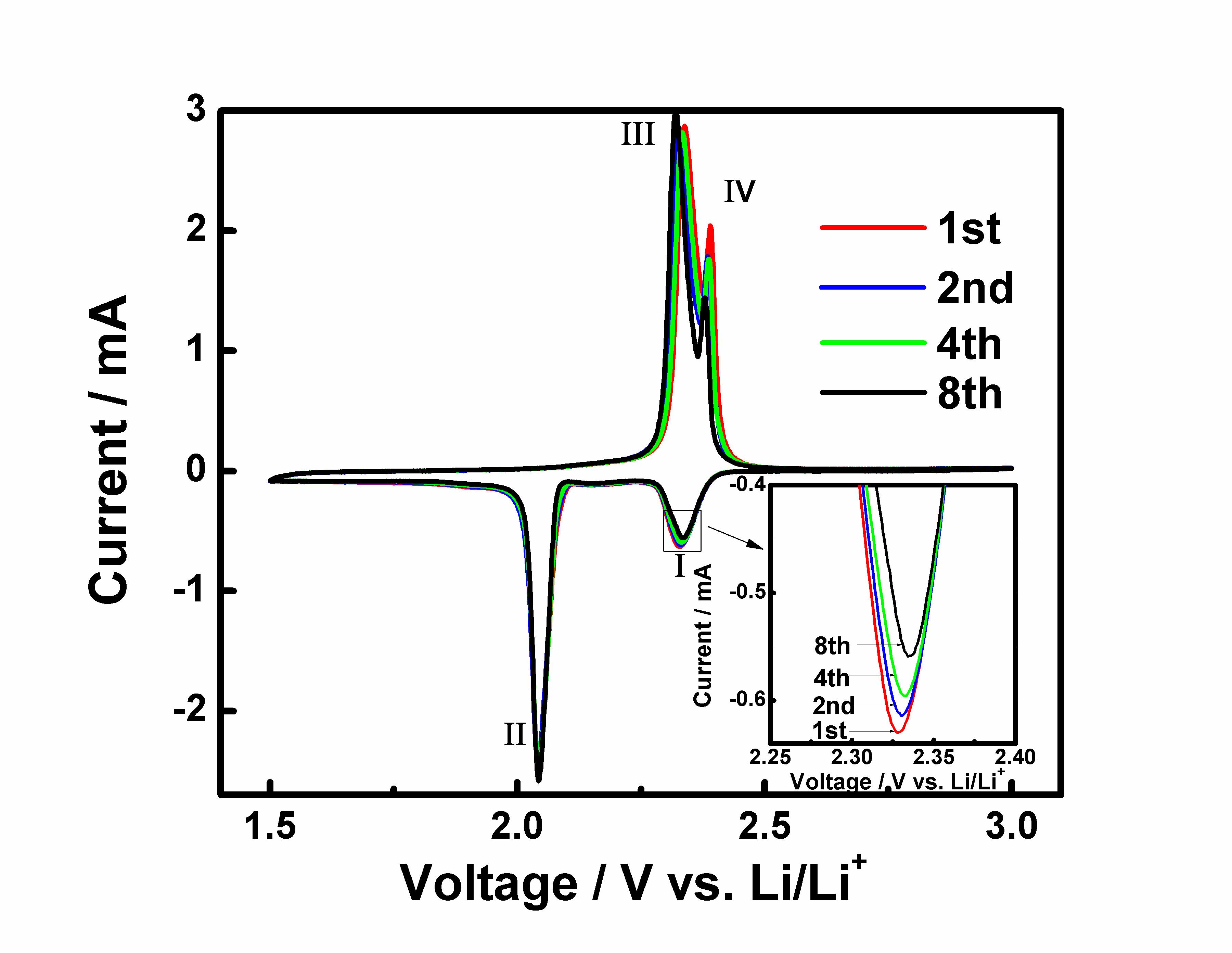


Supplementary Figure 5 | Cyclic voltammograms curves of the CNSMC/S-54 composite at 0.1 mv s^-1^.


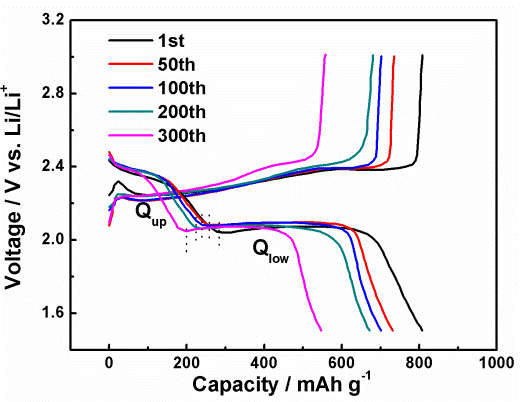


Supplementary Figure 6 | Galvanostatic charge-discharge curves of different cycles at 2 C rate. The Q_low_/Q_up_ ratios are 1.9, 1.9, 1.9, 1.8 and 1.8 in the 1st, 50th, 100th, 200th and 300th cycle, respectively. The high and stable Q_low_/Q_up_ ratio, which is close to the theoretical value of 2.0, confirms the adsorption ability of CNSMC to ensure the effective capture and utilization of the polysulfides.


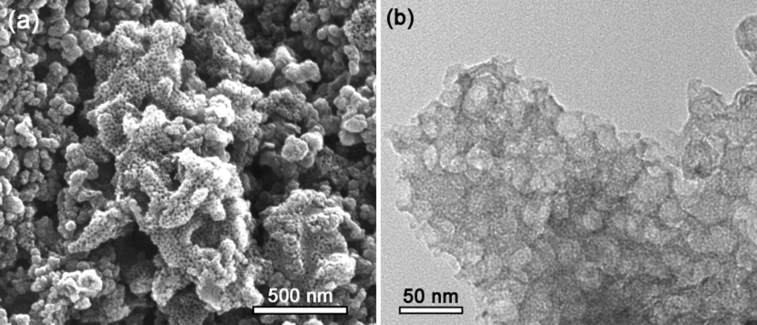


Supplementary Figure 7 | The morphology of the CNSMC in the CNSMC/S-54 composite cycled at 2 C rate for 100 cycles: **(a)** SEM image. **(b)** TEM image. Before the observation of the morphology changing of CNSMC, the cycled CNSMC/S composite electrode (charged to 3 V at 0.2 C in the last cycle) was first washed and soaked in the DME and DOL (1:1 v/v) for 2 h in a sealed glove box filled with Ar gas, then the electrode was heated at 280°C for 3h in a tube furnace under nitrogen flow to remove sulfur embedded in the carbon host.


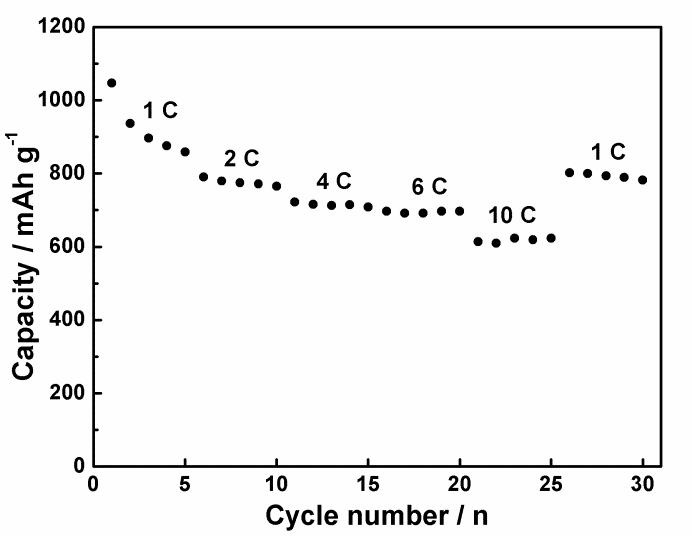


Supplementary Figure 8 | Rate performance of the CNSMC/S-54 composite.


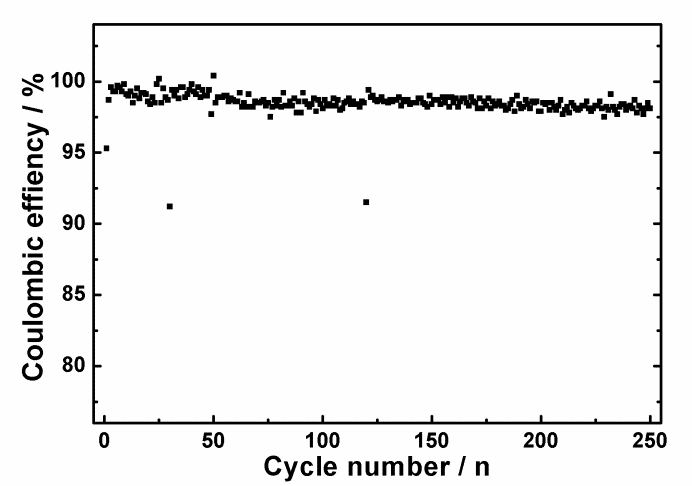


Supplementary Figure 9 | Coulombic efficiency of the CNMC/S-54 composite cycled at 2 C rate.


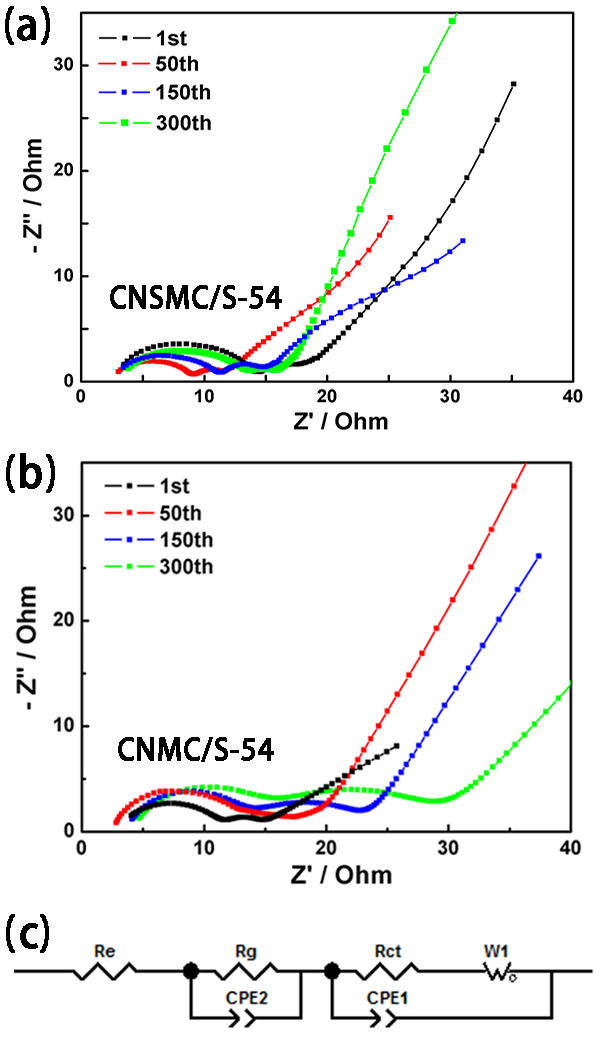


Supplementary Figure 10 | Nyquist plots of **(a)** the CNSMC/S-54 composite and **(b)** the CNMC/S-54 composites cycled at 2 C. **(c)** The equivalent circuit for the C/S composite electrodes.

**2. Supplementary Table**

Supplementary Table 1 | Results of elemental composition revealed by elemental analysis and XPS.

| Samples | C (wt %) | N (wt %) | S (wt %) | O (wt %) | H (wt %) |
| --- | --- | --- | --- | --- | --- |
| CNSMC  (by EA) | 75.1 | 5.0 | 4.6 | / | 1.7 |
| CNSMC  (by XPS) | 81.3 | 5.3 | 4.1 | 9.3 | / |
| CNSMC/S-54  (by EA) | 35.6 | 2.4 | 54.3 | / | 0.7 |
| CNMC  (by XPS) | 83.8 | 6.1 | 0.5 | 9.7 | / |

Supplementary Table 2 | Surface concentration (in at.%) of the oxygen and sulfur species for the CNSMC and CNSMC/S composite based on the high-resolution C1s and S2p XPS spectra.

| Sample | C=O | O-S | C-O | R-SO_3_ | R-S-O | S_8_/R-S | S^2-^ |
| --- | --- | --- | --- | --- | --- | --- | --- |
| CNSMC | 62.2 | 20.3 | 17.5 | 29.4 | 23.5 | 47.1 | / |
| CNSMC/S | 39.7 | 46.3 | 14.1 | 2.6 | 22.5 | 69.9 | 5.0 |

Supplementary Table 3 | Comparison of a set of high rate electrochemical performance of the carbon/sulfur composites for lithium-sulfur batteries.

| Composites | S content  (wt.) | Capacity based on the mass of the composite (mAh g^-1^) | Cycle number | Ref. |
| --- | --- | --- | --- | --- |
| CNSMC/S | 64% | 322 @ 2 C ^a)^ | 200 | Our work |
| CNT-S | 50% | 262 @ 0.9 C | 100 | 1 |
| HOPC/S | 50% | 241 @ 1 C | 100 | 2 |
| MWCNTs@S/NPC@PEG | 56.7% | 316 @ 2C | 50 | 3 |
| PANI@S/C | 43.7% | 260 @ 1 C | 100 | 4 |
| RGO-TG-S | 63% | 420 @ 0.95 C | 200 | 5 |
| G/SWCNT-S | 60% | 318 @ 1 C | 100 | 6 |

a) 1 C=1675 mAh g^-1^

**Supplementary References**

1. Zhou, G. *et al*. A flexible nanostructured sulphur-carbon nanotube cathode with high rate performance for Li-S batteries. *Energy Environ. Sci.* **5**, 8901-8906 (2012).
2. Ding, B. *et al*. Encapsulating sulfur into hierarchically ordered porous carbon as a high-performance cathode for lithium-sulfur batteries. *Chem. Eur. J.* **19**, 1013-1019 (2013).
3. Li, Z. *et al*. A dual coaxial nanocable sulfur composite for high-rate lithium-sulfur batteries. *Nanoscale* **6**, 1653-1660 (2014).
4. Li, G. C., Li, G. R., Ye, S. H. & Gao, X. P. A polyaniline-coated sulfur/carbon composite with an enhanced high-rate capability as a cathode material for lithium/sulfur batteries. *Adv. Energy Mater.* **2**, 1238-1245 (2012).
5. Li, N. *et al*. High-rate lithium–sulfur batteries promoted by reduced graphene oxide coating. *Chem. Commun.* **48**, 4106-4108 (2012).
6. Zhao, M. Q. *et al*. Graphene/single-walled carbon nanotube hybrids: one-step catalytic growth and applications for high-rate Li-S batteries. *ACS Nano* **6**, 10759-10769 (2012).
